# Supplementary material for: Implementing the Reactor Geometry in the Modeling of Chemical Bath Deposition of ZnO Nanowires
Source: Nanomaterials (Basel). 2022 Mar 24;12(7):1069. doi: 10.3390/nano12071069 (PMC9000409; doi:10.3390/nano12071069)
Supplement: Supplementary file 1 [file nanomaterials-12-01069-s001.zip › nanomaterials-1623025-supplementary.pdf]

Supporting Information

Implementing the Reactor Geometry in the Modeling of Chemical Bath Deposition of ZnO Nanowires

Clément Lausecker [1,2,3], Bassem Salem [2,*], Xavier Baillin [3] and Vincent Consonni [1,*]

[1] Université Grenoble Alpes, CNRS, Grenoble INP, LMGP, 38000 Grenoble, France; clement.lausecker@grenoble-inp.fr
[2] Université Grenoble Alpes, CNRS, CEA/LETI-Minatec, Grenoble INP, LTM, 38054 Grenoble, France
[3] Université Grenoble Alpes, CEA, LETI, 38054 Grenoble, France; xavier.baillin@cea.fr
* Correspondence: bassem.salem@cea.fr (B.S.); vincent.consonni@grenoble-inp.fr (V.C.)

Detailed proof of the dynamic growth model

To implement the reactor geometry in the modelization of the elongation process of ZnO NWs, we consider a reactor of finite height h, defined for $0 < z < h$, where an infinite substrate is placed at $z = 0$, as depicted in **Figure 1** in the main text. To establish the expression of the length of ZnO NWs under dynamic conditions – i.e. where the depletion of chemical reactants occurs, we apply Fick's second diffusion equation in one dimension on the concentration of Zn(II) ions [1]:

$$\frac{\partial C}{\partial t} = D\frac{\partial^2 C}{\partial z^2} \quad (4)$$

with the three following boundary conditions:

$$C(z, t = 0) = C_0 \quad (5)$$

$$\frac{dC(z = h, t)}{dz} = 0 \quad (6)$$

$$\frac{dC(z = 0, t)}{dz} = \frac{k_1 S}{D}\left(C(z = 0, t) - C_{eq}\right) \quad (7)$$

where C is the concentration of Zn(II) ions (m^{-3}) at a height z above the substrate and at a growth time t, C_{eq} is the equilibrium concentration of Zn(II) ions (m^{-3}), k_1 is the first-order reaction rate constant describing the crystallization process of ZnO (m.s^{-1}), S is the c-plane top surface area ratio and D is the diffusion coefficient of Zn(II) ions in aqueous solutions at a temperature T (m^2.s^{-1}).

Supporting Information

Equation 5 reports that the concentration of Zn(II) ions at a height z and at $t = 0$ is assumed constant wherever in the chemical bath and is defined as C_0. Equation 6 accounts for the finite height of the reactor. It is derived from Fick's first diffusion equation by assuming that the flux of Zn(II) ions at $z = h$ equals 0. Equation 7 relates to the consumption of the Zn(II) ions on the growth front located at the c-plane top facet of ZnO NWs. It is obtained by applying Fick's first diffusion equation at $z = 0$, while considering Zn(II) ions as the limiting reactants. It is also worth noticing that the development of the growth front with time along the z axis is neglected, and that S is considered as a constant parameter in a first approximation.

To solve this second-order partial differential equation, we apply the Laplace transform to Equation 4. Indeed, from its properties, we can write:

$$\frac{dc(z,p)}{dt} = pc(z,p) - C(z,t=0) \tag{7b}$$

where $c(z,p)$ is the Laplace transform of $C(z,t)$. Thus, if we apply the Laplace transform to Equation 4 and using Equation 5, we obtain:

$$\frac{\partial^2 c}{\partial z^2} - \frac{p}{D}c = \frac{-C(z,t=0)}{D} = \frac{-C_0}{D} \tag{8}$$

The temporal derivative is thus removed and we obtain an ordinary differential equation. The general solution of Equation 8 has the form:

$$c(z,p) = \frac{C_0}{p} + a_1 \exp\left(\sqrt{\frac{p}{D}}z\right) + a_2 \exp\left(-\sqrt{\frac{p}{D}}z\right) \tag{8b}$$

where a_1 and a_2 are constants to be determined. Applying the Laplace transform to Equations 6 and 7 we obtain:

$$\frac{dc(z=h,p)}{dz} = 0 \tag{8c}$$

$$\frac{dc(z=0,p)}{dz} = \frac{k_1 S}{D}\left(c(z=0,p) - \frac{C_{eq}}{p}\right) \tag{8d}$$

Using Equation 8c in Equation 8b, we deduce that:

$$a_1\sqrt{\frac{p}{D}}\exp\left(\sqrt{\frac{p}{D}}h\right) - a_2\sqrt{\frac{p}{D}}\exp\left(-\sqrt{\frac{p}{D}}h\right) = 0 \Leftrightarrow a_1 = a_2\exp\left(-2\sqrt{\frac{p}{D}}h\right) \quad (8e)$$

Furthermore, from Equation 8b we can note that:

$$c(z=0,p) = \frac{C_0}{p} + a_1 + a_2 \quad (8f)$$

$$\frac{dc(z=0,p)}{dz} = \sqrt{\frac{p}{D}}(a_1 - a_2) \quad (8g)$$

Then, reinjecting Equations 8f and 8g in Equation 8d we obtain:

$$\sqrt{\frac{p}{D}}(a_1 - a_2) = \frac{k_1 S}{D}\left(\frac{C_0 - C_{eq}}{p} + a_1 + a_2\right) \quad (8h)$$

From Equations 8e and 8h, we deduce a_1 and a_2:

$$a_1 = \frac{\frac{k_1 S}{D}(C_0 - C_{eq})}{p\left[\sqrt{\frac{p}{D}}\left(1 - \exp\left(2\sqrt{\frac{p}{D}}h\right)\right) - \frac{k_1 S}{D}\left(1 + \exp\left(2\sqrt{\frac{p}{D}}h\right)\right)\right]} \quad (8i)$$

$$a_2 = \frac{\frac{k_1 S}{D}(C_0 - C_{eq})}{p\left[\sqrt{\frac{p}{D}}\left(\exp\left(-2\sqrt{\frac{p}{D}}h\right) - 1\right) - \frac{k_1 S}{D}\left(\exp\left(-2\sqrt{\frac{p}{D}}h\right) + 1\right)\right]} \quad (8j)$$

Replacing Equations 8i and 8j in Equation 8b and rearranging, we get:

$$c(z,p) = \frac{C_0}{p} - \frac{k_1 S(C_0 - C_{eq})}{pD}\left[\frac{cosh\left(\sqrt{\frac{p}{D}}(h-z)\right)}{\frac{k_1 S}{D}cosh\left(\sqrt{\frac{p}{D}}h\right) + \sqrt{\frac{p}{D}}sinh\left(\sqrt{\frac{p}{D}}h\right)}\right] \quad (9)$$

It is worth noticing that at $z = 0$, Equation 9 simplifies into:

$$c(0,p) = \frac{C_0}{p} - \frac{(C_0 - C_{eq})}{p}\frac{1}{1 + \frac{\sqrt{pD}}{k_1 S}tanh\,(\sqrt{\frac{p}{D}}h)} \quad (10)$$

The concentration profile $C(z,t)$ of Zn(II) ions is in principle obtainable by applying the inverse Laplace transform to Equation 9 or 10. However, their forms are not trivial and this operation cannot be readily resolved analytically. Therefore, we perform the inverse Laplace transform within a numerical approach developed by Valsa *et al.* [2]. To efficiently apply this method, a freely accessible MATLAB

function, INVLAP, is used, which can readily provide numerical values at specific times t of the function $f(t)$ corresponding to the inverse Laplace transform of the input function $F(p)$ [3]. In the present case, we can obtain a graphical representation of the temporal evolution of the concentration of Zn(II) species at $z = 0$ by applying this function to Equation 10.

To deduce the time-dependent axial growth rate of the ZnO NWs denoted as R_c, we perform mass balance at the NW growth front, by considering that the flux of consumed Zn(II) ions is equal to the flux of Zn atoms crystallizing:

$$k_1 S\left(C(z=0,t) - C_{eq}\right) = R_c(t)\rho S \tag{10b}$$

where ρ is the atomic density of wurtzite ZnO that is equal to 4.20×10^{28} m^{-3}. Then we can deduce the relation:

$$R_c(t) = \frac{k_1\left(C(z=0,t) - C_{eq}\right)}{\rho} \tag{11}$$

The length of ZnO NWs denoted as L and its temporal dependence is, in turn, deduced by integrating Equation 11:

$$L(t) = \int_0^t R_c(\tau) d\tau \tag{12}$$

To compute this integral from discrete values of R_c taken at different times t, the trapezoidal rule is used to used (corresponding to the *cumtrapz* function in MATLAB), where a linear interpolation is performed between two consecutive values of R_c. Furthermore, the equilibrium concentration of Zn(II) ions C_{eq} can be typically determined from thermodynamic simulations, as detailed in Ref [4].

Supporting Information

Supporting figures

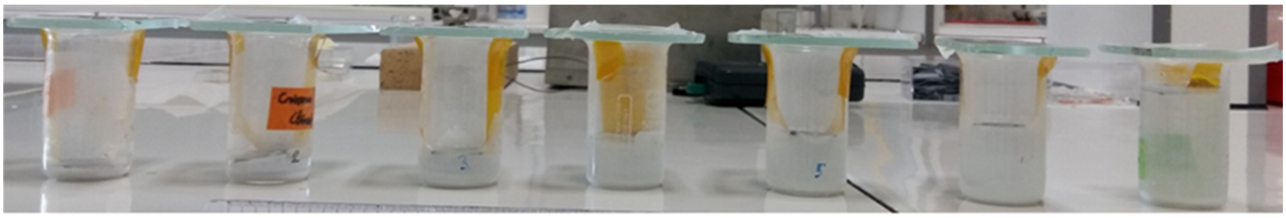

h = 0.5 cm **h** = 0.9 cm **h** = 1.2 cm **h** = 1.9 cm **h** = 2.1 cm **h** = 2.7 cm **h** = 4.0 cm

Figure S1. Pictures of the chemical baths containing 30 mM of $Zn(NO_3)_2$ and HMTA after 41 h at 90 °C with reactor heights *h* ranging from 0.5 to 4.0 cm. For the sake of consistency with the geometry used in the theoretical model, each sample of the series is placed at the top surface of the chemical bath, where the active side for the ZnO NW growth is facing downward.

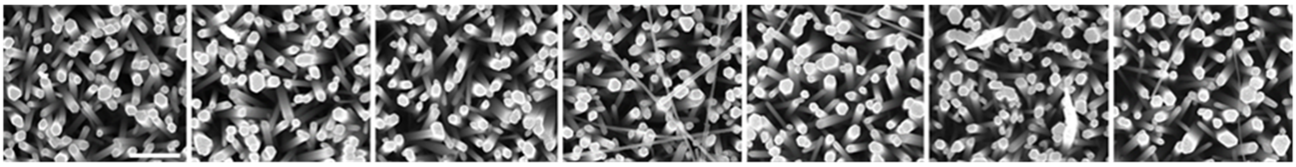

**h** = 0.5 cm	**h** = 0.9 cm	**h** = 1.2 cm	**h** = 1.9 cm	**h** = 2.1 cm	**h** = 2.7 cm	**h** = 4.0 cm
**S** = 0.29 ± 0.04	**S** = 0.28 ± 0.03	**S** = 0.32 ± 0.02	**S** = 0.29 ± 0.04	**S** = 0.30 ± 0.03	**S** = 0.33 ± 0.05	**S** = 0.29 ± 0.03

Figure S2. Top-view FESEM images of ZnO NWs grown by CBD at 90 °C with 30 mM of $Zn(NO_3)_2$ and HMTA for 41 h with reactor heights *h* ranging from 0.5 to 4.0 cm. The corresponding value of the *c*-plane top surface area ratio *S*, which was measured from such images, is also indicated for each sample. The scale bar represents 500 nm.

References

1. Fick, A. Ueber Diffusion. *Ann. Phys. Chem.* **1855**, *170*, 59–86, doi:10.1002/andp.18551700105.
2. Valsa, J.; Brančik, L. Approximate Formulae for Numerical Inversion of Laplace Transforms. *Int. J. Numer. Model. Electron. Netw. Devices Fields* **1998**, *11*, 153–166, doi:10.1002/(SICI)1099-1204(199805/06)11:3<153::AID-JNM299>3.0.CO;2-C.
3. Juraj. *Numerical Inversion of Laplace Transforms in Matlab*; version 1.0.0.0; The MathWorks, Inc.: Natick, MA, USA, 2011.
4. Lausecker, C.; Salem, B.; Baillin, X.; Consonni, V. Modeling the Elongation of Nanowires Grown by Chemical Bath Deposition Using a Predictive Approach. *J. Phys. Chem. C* **2019**, *123*, 29476–29483, doi:10.1021/acs.jpcc.9b08958.
